# Prognosis: the “missing link” within the CanMEDS competency framework

**DOI:** 10.1186/1472-6920-14-93

**Published:** 2014-05-13

**Authors:** Vincent Maida, Paul M Cheon

**Affiliations:** 1 University of Toronto, 101 Humber College Boulevard, 9th Floor, Toronto, Ontario M9V 1R8, Canada; 2 McMaster University, Toronto, Canada; 3 Division of Palliative Medicine, William Osler Health System, Toronto, Canada; 4 University of Toronto, 55 Centre Ave. Unit 1207, Toronto, Ontario M5G 2H5, Canada

**Keywords:** Prognosis, CanMEDS, Competency frameworks, Quoad vitam, Quoad sanantionem, Foreseeing, Foretelling

## Abstract

**Background:**

The concept of prognosis dates back to antiquity. Quantum advances in diagnostics and therapeutics have relegated this once highly valued core competency to an almost negligible role in modern medical practice. Medical curricula are devoid of teaching opportunities focused on prognosis. This void is driven by a corresponding relative dearth within physician competency frameworks. This study aims to assess the level of content related to prognosis within CanMEDS (Canadian Medical Education Directives for Specialists), a leading and prototypical physician competency framework.

**Methods:**

A quantitative content analysis of CanMEDS competency framework was carried out to measure the extent of this deficiency. Foxit Reader 5.1 (Foxit Corporation), a keyword scanning software, was used to assess the CanMEDS 2005 framework documents of 29 physician specialties and 37 subspecialties across the seven physician roles (medical expert, communicator, collaborator, manager, health advocate, scholar, and professional). The keywords used in the search included prognosis, prognostic, prognosticate, and prognostication.

**Results:**

Of the 29 specialties six (20.7%) contained at least one citation of the keyword “prognosis”, and one (3.4%) contained one citation of the keyword “prognostic”. Of the 37 subspecialties, sixteen (43.2%) contained at least one citation of the keyword “prognosis”, and three (8.1%) contained at least one citation of the keyword “prognostic”. The terms “prognosticate” and “prognostication” were completely absent from all CanMEDS 2005 documents. Overall, the combined citations for “prognosis” and “prognostic” were linked with the following competency roles: Medical Expert (80.3%), Scholar (11.5%), and Communicator (8.2%).

**Conclusions:**

Given the fundamental and foundational importance of prognosis within medical practice, it is recommended that physicians develop appropriate attitudes, skills and knowledge related to the formulation and communication of prognosis. The deficiencies within CanMEDS, demonstrated by this study, should be addressed in advance of the launch of its updated version in 2015.

## Background

The birth of prognosis, using clinical signs, dates back to ancient Sumerian civilization circa 2,000 BC [1]. Hippocrates (460 BC – 370 BC) advanced the domain of prognosis by using combinations of symptoms and clinical signs to predict outcomes. Hippocratic prognostication also took into account certain environmental factors and patient characteristics but did not take into account the patient’s diagnosis. During the era of Hippocrates the core competencies of a physician consisted of diagnosis, therapeutics, and prognosis. However, the paucity of effective diagnostic and therapeutic modalities rendered the ability to prognosticate the most important role of a physician [1-3]. Conceptually, Hippocrates described prognosis as a two dimensional construct: *quoad vitam* (predictions about survival and life expectancy) and *quaod sanantionem* (predictions about healing and restoration of function) [2,3].

Over the past century monumental advances in diagnostic and therapeutic modalities have led to the marginalization of prognosis as a core competency. This trend is reflected by its withering presence within medical literature. The original comprehensive textbook of medicine, written by Sir William Osler (1849 to 1919), “*The Principles and Practice of Medicine*” published twenty-two editions between 1892 and 1988. Osler’s textbook described each disease under seven categories: etiology, presentation, pathology, diagnosis, therapy, prognosis, and complications. Osler’s prognostic formulations took into account symptoms, signs, and diagnostic criteria. Review of a leading contemporary forerunner, “*Harrison’s Principles of Internal Medicine*”, 12^th^ edition, revealed that only 27% of the diseases had dedicated sections discussing prognosis [4].

The post Osler era has witnessed the relegation of prognosis to a negligible level of importance. This has occurred in tandem with decreased emphasis, if not denial, of the natural history of disease. In modern times physicians are trained and socialized with a nearly singular objective, to cure. Anything less is regarded as a failure. As a result, there exists a tendency to avoid consideration of prognosis as this would seem to promote nihilism and negativism. Thus, the need to render a prognosis has been deemed redundant, and has led to a noticeable void within medical curricula. Moreover, competency frameworks, which are the main drivers of medical curricular development scarcely touch upon topics related to prognosis. The implications of this trend for patient, family, and society are immense, not the least of which is the tendency for physicians to excessively offer late stage interventions to patients within the final phases of their lives, thus, denying them the opportunity to be managed more appropriately and effectively in a conservative palliative mode of care. In addition, when prognosis is not disclosed, treatment decisions tend to be more paternalistically driven by physicians, often associated with vested interests, rather than being truly patient-centered [4].

At the vanguard of a fledgling renaissance for prognosis in medical practice has been Dr. Nicholas A. Christakis. His award winning book “Death Foretold-Prophecy and Prognosis in Medical Care”, published in 1999, eloquently outlines the deficiencies related to prognosis in clinical care and research together with the serious implications for patients, families and society. Dr. Christakis passionately urges that prognosis be restored as a core competency. His thesis, built upon Hippocratic principals, posits that the process of prognosis comprises two basic components, namely, *foreseeing* (computing and formulating the prognosis) and *foretelling* (disclosing and communicating the prognosis). Hence, prognosis may be viewed as both a science (foreseeing) and an art (foretelling) [4]. Thus, a complete and comprehensive approach to prognosis must involve both components.

The impact of Christakis’s work is evidenced by the observation that over the past decade there has been a steep rise in the number of publications related to objective prognostic factors for both quoad vitam and quoad sanantionem. For the purposes of quoad vitam, the TNM (Tumor, Node, Metastasis) anatomic cancer classification system [5], and actuarial data, outlined in Surveillance, Epidemiology, and End-Results program of the National Cancer Institute (SEER) are predictive of long-term survival in patients with newly diagnosed cancer [6]. In advanced cancer Palliative Performance Scale scores (PPS) [7,8], Palliative Prognostic Index (PPI) [9], and Palliative Prognostic Score (PaP) [10] are predictive of short-term survival. In the Intensive Care Unit setting, the Acute Physiology and Chronic Health Evaluation Score (APACHE) [11] and Simplified Acute Physiology Score (SAPS) [12] correlate with survival. Moreover, recent research is also demonstrating the potential of novel prognostic factors such as tumour-related factors (cytogenic and molecular markers) and inflammatory markers [13]. The occurrence of sentinel events in dementia cases such as dysphagia, sepsis, and pressure ulcers also portend a shortened life expectancy [14,15]. Examples of assessment tools that facilitate quoad sanantionem include the FIM (Functional Independence Measure) [16,17] being predictive of neurologic recovery in stroke patients, and PPS being predictive of pressure ulcer healing [18]. However, despite the existence of data on such factors, instruments, and models, the process of knowledge translation remains laggard, rendering physician utilization low.

A paradigm shift to a competency-based approach in medical education is the result of public outcry for improved quality, comprehensiveness, and accountability [19,20]. One of the original competency frameworks was created during the early 1990’s by the Royal College of Physicians and Surgeons of Canada (RCPSC), and was called the “Canadian Medical Education Directives for Specialists” (CanMEDS). Since 1993, CanMEDS has gone through four major development phases. The current 2005 version is based upon the seven core roles of a physician: Medical Expert, Communicator, Collaborator, Manager, Health Advocate, Scholar, and Professional. The Medical Expert role is regarded as the central role as it represents a balanced integration of the other six roles. The CanMEDS framework has been incorporated into medical undergraduate and postgraduate programs throughout Canada. Moreover, CanMEDS is foundational in defining educational objectives and outcomes of all medical specialties and subspecialties recognized by the RCPSC. CanMEDS has been recognized as a prototypical competency framework having been adopted, adapted, and modelled internationally. Currently, CanMEDS is in the midst of a three-year reform process that is scheduled to be finalized in 2015 [21].

This study aims to quantify the level of content related to the domain of prognosis within CanMEDS 2005. It is hoped that by highlighting any deficiencies, attention may be focused on recognizing and restoring prognosis as a core physician competency, both from the perspective of a scholar (foreseeing) and a communicator (foretelling).

## Methods

This study employed the methodology of quantitative content analysis [22]. The entire compilation of CanMEDS 2005 text documents were accessed on the website of the Royal College of Physicians and Surgeons of Canada [21] on July 31, 2013. The online documents found under “Information by Discipline” were subjected to Foxit Reader 5.1 (Foxit Corporation), a proprietary scanning software that locates the presence of keywords within text-based documents [23]. All documents pertaining to 29 medical specialties and 37 subspecialties, across the seven physician roles (medical expert, communicator, collaborator, manager, health advocate, scholar, and professional) were subjected to the Foxit Reader 5.1 scan. The keywords used in the search included prognosis, prognostic, prognosticate, and prognostication. The presence of the particular keywords were then manually entered onto Microsoft Excel 2007 spreadsheets in order to tabulate the frequency of occurrences per specialty and subspecialty, as well as categorizing them as to which of the physician roles they were linked with. No human material or human data were used in this research.

## Results

Among the 29 specialties there were 20 citations of the keyword “prognosis” and one for “prognostic”. (Tables 1 and 2). Neurology accounted for 13 of the 20 (65%) citations for the keyword “prognosis”. Of the 29 specialties six (20.7%) contained at least one citation of the keyword “prognosis”, and one (3.4%) contained one citation of the keyword “prognostic” (Tables 1 and 2). The combined citations for “prognosis” and “prognostic” among specialties were linked with the following competency roles: Medical Expert (81%), Scholar (14.3%), and Communicator (4.7%) (Table 1).

**Table 1:** Specialties with citations of keywords “prognosis” or “prognostic” (n = 7)

**Specialty**	**Keyword**		**Competency domain**		
	**Prognosis (Number of citations)**	**Prognostic (Number of citations)**	**Medical expert (Number of citations)**	**Communicator (Number of citations)**	**Scholar (Number of citations)**
Dermatology	1	0	1	0	0
Medical Genetics	2	0	1	1	0
Neurology	13	0	10	2	1
Neurosurgery	1	0	1	0	0
Obstetrics & Gynecology	2	0	2	0	0
Otolaryngology-Head & Neck Surgery	1	0	1	0	0
Plastic Surgery	0	1	1	0	0
**Total number of Specialties**	**6**	**1**	**7**	**2**	**1**

**Table 2:** Subspecialties with citations of keywords “prognosis” or “prognostic” (n = 19)

**Subspecialty**	**Keyword**		**Competency domain**		
	**Prognosis (Number of citations)**	**Prognostic (Number of citations)**	**Medical expert (Number of citations)**	**Communicator (Number of citations)**	**Scholar (Number of citations)**
Cardiology (Pediatric)	1	0	1	0	0
Cardiology (Adult)	1	0	1	0	0
Critical Care Medicine (Pediatric)	2	0	1	0	1
Critical Care Medicine (Adult)	1	0	0	0	1
Developmental Pediatrics	1	0	0	0	1
Forensic Psychiatry	1	0	0	0	1
Gastroenterology	1	0	1	0	0
General Surgical Oncology	14	0	14	0	0
Geriatric Medicine	0	1	1	0	0
Gynecologic Oncology	1	0	1	0	0
Gynecologic Reproductive Endocrinology & Infertility	1	0	0	0	1
Hematology	0	2	2	0	0
Maternal Fetal Medicine	1	0	0	0	1
Medical Oncology	0	3	2	1	0
Pain Medicine	5	0	5	0	0
Pediatric Hematology & Oncology	1	0	0	1	0
Respirology (Pediatric)	1	0	1	0	0
Respirology (Adult)	1	0	1	0	0
Thoracic Surgery	1	0	1	0	0
**Total number of Subspecialties**	**16**	**3**	**13**	**2**	**6**

**Table 3:** Specialties & Subspecialties with no citations of keywords “prognosis” or “prognostic”

**Specialty N=22**	**Subspecialty N=18**
	Adolescent Medicine
Anatomic Pathology	Child & Adolescent Psychiatry
Anesthesiology	Clinical Immunology & Allergy
Cardiac Surgery	Clinical Pharmacology & Toxicology
Diagnostic Radiology	Colorectal Surgery
Emergency Medicine	Endocrinology & Metabolism
General Pathology	Forensic Pathology
General Surgery	General Internal Medicine
Hematologic Pathology	Geriatric Psychiatry
Internal Medicine	Infectious Diseases
Medical Biochemistry	Neonatal Perinatal Medicine
Medical Microbiology	Nephrology
Neuropathology	Neuroradiology
Nuclear Medicine	Occupational Medicine
Ophthalmology	Pediatric Emergency Medicine
Orthopedic Surgery	Pediatric Radiology
Pediatrics	Pediatric Surgery
Physical Medicine & Rehabilitation	Rheumatology
Psychiatry	
Public Health and Preventive Medicine	
Radiation Oncology	
Urology	
Vascular Surgery	

Among the 37 subspecialties there were 34 citations of the keyword “prognosis” and six for “prognostic” (Table 2). General Surgical Oncology accounted for 14 of the 34 (41.2%) citations for the keyword “prognosis”. Of the 37 subspecialties, sixteen (43.2%) contained at least one citation of the keyword “prognosis”, and three (8.1%) contained at least one citation of the keyword “prognostic” (Table 2). The combined citations for “prognosis” and “prognostic” among subspecialties were linked with the following competency roles: Medical Expert (80%), Scholar (15%), and Communicator (5%) (Table 2).

Table 3 summarizes the 22 specialties and the 18 subspecialties that were found to have no citations of the keywords “prognosis” or “prognostic”. The following keywords had zero citations: prognosticate, prognostication, foresee, foretell, and survival estimate. Vascular Surgery was found to have one citation with the keyword life expectancy.

## Discussion

CanMEDS 2005 is deficient in its content pertaining to the domain of prognosis. Only 26 of the 66 (39.4%) combined medical specialties and subspecialties were found to have at least one citation related to the keywords prognosis and prognostic. Overall, there were only 61 citations for the keywords prognosis and prognostic. The distribution of the total citations was skewed as 13 (21.3%) were associated with the specialty of Neurology, and 14 (22.9%) were associated with the subspecialty of General Surgical Oncology. The deficiencies within CanMEDS are further emphasized by the observation that the following specialties and subspecialties had zero citations associated with prognosis despite being heavily involved in the management of terminally ill patients: Radiation Oncology, Internal Medicine, General Internal Medicine, General Surgery, Colorectal Surgery, and Nephrology. Moreover, the following specialties and subspecialties had zero citations associated with prognosis despite being heavily involved in the management of patients who are receiving rehabilitative services: Orthopedic Surgery, Physical Medicine and Rehabilitation, and Rheumatology. These findings are consistent with published data that reports more than 90% of physicians are “reluctant to make predictions” about a patient’s illness [4], and even when provided with objective prognostic estimates, discuss it with patients and/or substitute decision makers 15% of the time [24]. Failure to disclose prognoses to terminally ill patients is emerging as a medical-legal issue [25]. In a number of cases where prognostic information was not disclosed, the rulings were in favour of the plaintiff [26]. In the United States of America, the states of California and New York have “Palliative Care Information Acts” that mandate the disclosure of prognostic information by healthcare professionals in the setting of terminal illness [27]. Moreover, failure to prognosticate is associated with numerous significant concerns that apply to the complete spectrum of bioethics [4,28].

Most of the keyword citations in this study were linked to the competency role of “Medical Expert” (80.3%), while 11.6% were linked with the “Scholar” role, and 8.1% were linked with the “Communicator” role. Although the CanMEDS “Medical Expert” role is an integration of the six other roles, the discussion of a complex and multifaceted issue such as prognosis requires detailed discussion under more than one competency role. Thus, competency frameworks such as CanMEDS must acknowledge that a comprehensive and effective approach to prognosis may only be achieved by articulating the need for objective computation of prognostic estimates through the “Scholar” role, together with skillful disclosure to the patient and/or substitute decision maker through the “Communicator” role. In other words, the “Scholar” role promotes “Foreseeing”, while the “Communicator” role promotes “Foretelling”. Foreseeing may be regarded as a series of scholarly activities that begin with the knowledge translation of available data on prognostic factors, instruments, and tools, followed by the computation of a prognostic estimate. Successful Foretelling is dependent on the physician being effective at “breaking bad news” [29]. Thus, physicians must develop skills to deliver prognostic estimates in a gentle and sensitive, yet, confident manner. In addition, physicians must be able to detect emotions and respond empathetically, while being able to gauge the patient’s understanding of the delivered prognostic estimate [30]. Given the inherent uncertainty and probabilistic nature, prognostic quotations should never be stated in exact terms, but rather as ranges (days to weeks, weeks to months, months to years), or as median survival [1,4].

**Figure 1:**
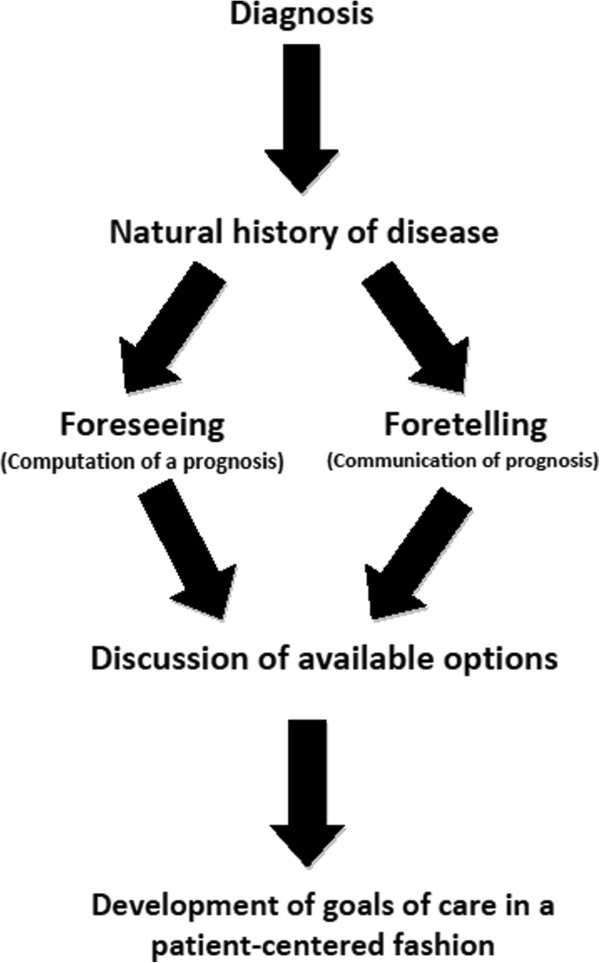
A paradigm for incorporating prognosis into patient-centered decision making.

A useful paradigm for incorporating prognosis into patient-centered decision making is shown in Figure 1. The process begins with a discussion about diagnosis. This is followed by a discussion pertaining to the natural history of the particular disease or affliction. The physician then computes a prognostic estimate and then communicates it to the patient. This is followed by a discussion of the available options along with success rates, benefits, risks, and burdens. Finally, the patient arrives at a decision that is consistent with his/her preferences, wishes, and values.

## Conclusions

The provision of a truly patient-centered and ethically sound approach to healthcare is dependent upon prognosis being both formulated and communicated to the patient and/or substitute decision maker. This study has demonstrated deficient levels of content related to the domain of prognosis within the CanMEDS 2005 competency framework. It is recommended that in advance of the launching of CanMEDS 2015, reforms occur that enhance content pertaining to prognosis within the outcome documents of all medical specialties and subspecialties. In order to hold true to the Hippocratic definition of prognosis as a two dimensional construct, both *quoad vitam* and *quoad sanantionem* should be discussed. It is also recommended that foreseeing should be discussed within the context of the Scholar role while foretelling should be discussed within the context of the Communicator role. Ultimately, increased attention to prognosis within CanMEDS and other competency frameworks carries the potential to promote medical curricular reform, thus moving towards restoring prognosis as a core physician competency.

## Competing interests

The authors declare that they have no competing interests.

## Authors’ contributions

VM and PMC designed the study. PMC conducted all of the document scanning, data collation, and analysis. VM drafted the manuscript. VM and PMC read and approved the final manuscript.

## Pre-publication history

The pre-publication history for this paper can be accessed here:

http://www.biomedcentral.com/1472-6920/14/93/prepub
